# Ageing, disability and long-term care

**DOI:** 10.1186/1472-6963-11-S1-A13

**Published:** 2011-10-19

**Authors:** D Nogueira, E Reis, R Atalaia, P Raposo, R Serrasqueiro

**Affiliations:** 1 ISCTE-IUL UNIDE-IUL, Lisbon, 1649, Portugal; 2 ESSA Escola Superior de Saúde de Alcoitão, 2765, Estoril, Portugal; 3 Pulido Valente Hospital, Lisbon, 1750, Portugal

## Introduction

The probability of entering a nursing home is not the same for everyone. Age, being a woman, and living alone are all risk factors. These risk factors are also interrelated: women have a higher probability of entering a nursing home, largely because they live longer and are more likely to live alone during their last years.

Disability also increases the risk of entering a nursing home. The more assistance a person needs, the greater the risk of being institutionalized. Certain types of disabilities have also been associated with increased risk of nursing home admission. For example, people who are cognitively impaired have a greater probability of entering a nursing home because cognitive disorders usually require constant supervision.

The existence of informal caregivers reduces the risk of nursing home use. Many studies have closely examined the role of informal caregivers in reducing the probability of nursing home admission. These studies also show that the attitude families have towards nursing homes influences the institutionalization of a family member.

Most users of nursing-home care have multiple and severe impairments, and they are dependent for care in more than three activities of daily living. In relation to general health, the institutionalized elderly have a multiple variety of diagnoses. The more common are diseases of the circulatory system, along with the mental disorders associated with that problem. Those with dementia normally have behavioral problems.

Many recent studies have estimated that nursing home utilization rates may be declining, and that the decline will continue to occur even though the number of very old is increasing. However, it is difficult to find a simple reason to explain trends in institutionalization. Declining utilization rates can be better explained by a reduction of supply, rather than by a decrease in the of prevalence of severe disability.

Financing of nursing home care – and who pays for it – is a particularly salient issue with regard to public expenses. Admission control, and payment for complexity, thus become priorities for those who manage this type of long-term care. The group includes family members, providers, and care-payers. In Portugal, the payment for long-term care is a co-payment divided between the elderly, or their family members, and the state.

## Method

This paper describes the profile of Portuguese elderly admitted to nursing homes that belong to the non-profit sector.

The main objective of the research was to describe the elderly in terms of their general health conditions and motor and cognitive impairment, and to determine the levels of disability (clinical complexity) that were associated with the different levels of utilization of resources. Recognizing the diversity of the elderly with functional impairments is essential in order to develop policies that are responsive to the range of needs that exist among the elderly.

To establish the disability profile, approximately 200 elderly, residing in various nursing homes, were evaluated. Areas considered were general health, frailty, risk and consequences of falls, swallowing disorders, communication profile, performance of the activities of daily living, cognitive ability levels and depression. The instruments used were a general questionnaire, the Lawton scale, Katz Index, Barthel Index, Mini-Mental State Examination, Geriatric Depression Scale, Verbal Association Test, Braden Scale, and a Swallowing Assessment. In addition, there was also a frailty indicator.

In order to identify the resources that were utilized for the different levels of care, a questionnaire was given to the managers of the institutions.

## Results and conclusions

Based on the different instruments used, and the functional impairments found, different levels of complexity were discovered among nursing home residents. However, the payment of care remains almost the same among these residents, except for small differences that do not reflect levels of assistance.

The range of disability within the institutionalized elderly is extremely wide. Service programs and financing mechanisms must reflect the vast range of service needs which the disabled elderly require. For policy purposes, it is generally useful to think of the disabled elderly in three large groups:

1. Elderly with mild impairments who do not require the active help of others.

2. Elderly with moderate impairments who do not need 24-hour assistance.

3. Elderly with severe limitations who require 24-hour intensive levels of care.

The most important conclusion the study reached is that the institutionalized elderly, since they have different levels of dependency, need flexible responses. The policies adopted, the planning of services, and their provision must be related to the assistance the institutionalized elderly need. The frequency and intensity of care is diverse, and these factors have a different impact on public and family spending that must be considered along with a systematic assessment of the dependency levels of the elderly.

